# Histopathological Analysis of Thrombi in Acute Ischemic Stroke: An Exploratory Study of Thrombus Composition and CD34-Positive Endothelial Cells

**DOI:** 10.3390/diagnostics16091390

**Published:** 2026-05-03

**Authors:** Sena Aksoy, Atay Vural, İbrahim Kulaç, Hatem Hakan Selçuk, Ali Burak Kızılırmak, Yasemin Gürsoy Özdemir, Bayram Yılmaz

**Affiliations:** 1 Department of Neurology, Bakırköy Prof. Dr. Mazhar Osman Mental Health and Neurological Diseases Training and Research Hospital, University of Health Sciences, Istanbul 34147, Turkey; 2 Department of Neurology, Faculty of Medicine, Istanbul Aydın University, Istanbul 34295, Turkey; 3 Department of Neurology, Faculty of Medicine, Koç University, Istanbul 34010, Turkey; 4 Research Center for Translational Medicine, Koç University, Istanbul 34010, Turkey; 5 Department of Pathology, Faculty of Medicine, Koç University, Istanbul 34010, Turkey; 6 Department of Neuroradiology, Bakırköy Dr. Sadi Konuk Training and Research Hospital, University of Health Sciences, Istanbul 34147, Turkey; 7 Department of Neuroradiology, Acıbadem Atasehir Hospital, Istanbul 34642, Turkey; 8 Department of Physiology, Faculty of Medicine, Yeditepe University, Istanbul 34755, Turkey; 9 Department of Physiology, Faculty of Medicine, Dokuz Eylül University, Izmir 35340, Turkey

**Keywords:** acute ischemic stroke, thrombus, mechanical thrombectomy, CD34, fibrin, red blood cell

## Abstract

**Background/Objectives:** Mechanical thrombectomy (MT) enables direct examination of the retrieved thrombus in acute ischemic stroke. Thrombus composition may influence treatment outcomes and reflect underlying stroke mechanisms. This study aimed to analyze thrombus histological composition and CD34-positive endothelial cells and evaluate their association with clinical and radiological characteristics. **Methods:** Fifty-six patients with acute ischemic stroke who underwent MT were included. Thrombi were classified as fibrin-dominant or red blood cell (RBC)-dominant using hematoxylin–eosin staining (H&E). Endothelial cells were identified via CD34 immunostaining. Associations between thrombus composition, procedural variables, imaging findings, and clinical outcomes were analyzed. **Results:** Forty-one (73.2%) thrombi were fibrin-dominant, and 15 (26.8%) were RBC-dominant. Fibrin-dominant thrombi were significantly associated with more distal occlusions (*p* = 0.027) and with the use of stent-retrievers (*p* = 0.045). RBC-dominant thrombi were more frequently associated with the hyperdense artery sign (HAS) (*p* = 0.015). CD34-positive staining correlated with shorter symptom-to-door (*p* = 0.017) and symptom-to-puncture times (*p* < 0.001). Endothelial ingrowth was more common in thrombi from proximal occlusions (*p* = 0.017). No significant associations were observed between thrombus composition and recanalization success, number of passes, or functional outcomes. The association between RBC-dominant thrombi and HAS supports the potential role of imaging markers in predicting thrombus composition prior to intervention. In addition, the presence and distribution of CD34-positive endothelial cells in relation to time intervals and occlusion location may reflect dynamic processes such as thrombus organization and vessel wall interaction. **Conclusions:** These findings highlight the heterogeneous nature of thrombus in acute ischemic stroke. Further studies are needed to clarify the biological and clinical implications of these observations.

## 1. Introduction

The widespread use of mechanical thrombectomy has enabled direct retrieval and analysis of thrombi responsible for acute ischemic stroke. Thrombi mainly consist of red blood cells, platelets, fibrin, and leukocytes and display distinct RBC-rich and platelet-fibrin–rich regions that can be identified using H&E staining [1]. The composition of retrieved thrombi may influence treatment outcomes and reflect underlying stroke mechanisms [2]. This advancement has facilitated numerous studies aimed at investigating potential correlations between thrombus composition and stroke etiology, as well as procedural outcomes [3,4,5,6].

CD34, a transmembrane glycoprotein expressed on hematopoietic stem cells and circulating endothelial cells, has drawn increasing interest in thrombus biology and ischemic stroke. CD34-positive endothelial progenitor cells are thought to contribute to neovascularization and endothelial repair following vascular injury—processes that are particularly relevant in the context of ischemic events [7]. The role of endothelial cells within thrombi may suggest a potential link between thrombus organization, vessel wall interaction, and time-dependent biological changes [8].

This study aims to investigate the histological composition of thrombi retrieved during mechanical thrombectomy in patients with acute ischemic stroke and assess the potential associations between thrombus composition, radiological features, and clinical outcomes, with a particular focus on the presence and distribution of endothelial cells within the thrombi.

## 2. Materials and Methods

### 2.1. Patient Selection

The patients admitted to the hospital with the diagnosis of acute ischemic stroke and treated with mechanical thrombectomy with/without iv tPA were included in this study. Intravenous tPA was administered to eligible patients after careful evaluation of indications and contraindications, such as anticoagulant therapy and a large ischemic core. The study included patients with ischemic stroke in the anterior circulation involving ICA, M1, or dominant M2 occlusions.

Ethical approval for this study was obtained from the Yeditepe University Clinical Research Ethics Committee (Approval number: 1212). Informed consent was obtained from all subjects included in the study.

### 2.2. Clinical Information

Demographic data such as age and sex, along with detailed clinical information including National Institutes of Health Stroke Scale (NIHSS) scores and the presence of comorbidities, were recorded for all patients. Time intervals were documented, including symptom-to-door, symptom-to-puncture, and puncture-to-recanalization times.

Clinical outcomes were assessed using NIHSS scores at 24 h and at discharge, and the modified Rankin Scale (mRS) at 90 days. Stroke etiology was classified according to the TOAST (Trial of ORG 10172 in Acute Stroke Treatment) criteria.

### 2.3. Endovascular Procedures and Neuroimaging

During mechanical thrombectomy, the treatment approach was selected based on factors such as occlusion site, vascular anatomy, and thrombus characteristics by a neurointerventionalist. Procedural data, including the number of passes, technique employed, and post-procedural recanalization scores based on the modified Thrombolysis in Cerebral Infarction (mTICI) scale, were collected.

The presence of the hyperdense artery sign was assessed on initial non-contrast cranial CT scans. Additionally, the presence of intracranial hemorrhage was evaluated on follow-up CT imaging at 24 h using the ECASS (European Cooperative Acute Stroke Study) criteria.

### 2.4. Thrombus Analysis

Retrieved thrombus samples were gently rinsed with saline and fixed in 10% formaldehyde solution for a period ranging from 24 h to a maximum of seven days. Following fixation, the samples were processed for paraffin embedding, sectioned, and stained with hematoxylin and eosin for the evaluation of fibrin and red blood cells. Histopathological examination was used to quantify RBC and fibrin content. For each thrombus, one representative section was stained.

All thrombus samples were fixed in 10% formaldehyde solution. Following fixation, tissue processing was performed using an automated tissue processor (Tissue-Tek VIP 6 AI, Sakura Finetek, Torrance, CA, USA), employing a standard formaldehyde–ethanol–xylene–paraffin protocol. Paraffin-embedded tissues were sectioned at a thickness of 4 μm and mounted on glass slides. Hematoxylin and eosin (H&E) staining was conducted following standard procedure. H&E-stained slides were then reviewed microscopically, and representative images were captured for further analysis.

CD34 immunohistochemical staining was performed to identify endothelial cells within the thrombus. An automated staining system (Dako Omnis, Agilent Technologies, Santa Clara, CA, USA) was used for the procedure. Tissue sections of 3 μm thickness were prepared from paraffin blocks and mounted on glass slides. Deparaffinization was carried out at 25 °C using Clearify Cleaning Agent (Agilent Technologies), followed by a 5 s rinse with distilled water. Antigen retrieval was performed by incubating the sections at 97 °C for 30 min in EnVision FLEX Target Retrieval Solution (Agilent Technologies), High pH (50×, pH 9; Dako Omnis). Subsequently, the sections were incubated with anti-CD34 antibody (Dako Omnis, GA63261) for 20 min and then rinsed with wash buffer for 2 min. This was followed by sequential incubation with EnVision FLEX Peroxidase-Blocking Reagent (3 min) and Mouse Linker (10 min), with wash buffer applied after each step. Next, sections were incubated with EnVision FLEX/HRP polymer for 20 min and washed three times with wash buffer (2 min each), followed by distilled water. Visualization was achieved using EnVision FLEX DAB chromogen for 20 min, followed by washing with DAB substrate buffer for 5 min and wash buffer for 2 min. Counterstaining was performed with hematoxylin (Dako Omnis) for 4 min, followed by rinses in distilled water (2 min) and wash buffer (2 min). After staining, slides were scanned to produce digital images for microscopic evaluation.

### 2.5. Image Analysis

Orbit (version 3.64) and Fiji (version 1.0) programs were used for image analysis. The analysis of thrombi stained with H&E was performed by using a color-based segmentation method to determine the relative quantitative fraction of RBC and fibrin in both Orbit and Fiji.

For quantitative analysis of immunohistochemical staining with CD34, five sections with ×20 magnification were chosen for each slide, and cell counting was performed manually by using the Fiji plugin CellCounter (National Institutes of Health, Bethesda, MD, USA). Field selection was based on areas demonstrating adequate tissue preservation and staining quality while avoiding regions with significant artifacts.

Although formal inter-observer variability analysis was not performed, all evaluations were conducted under standardized conditions by experienced investigators throughout the analysis to minimize variability.

### 2.6. Statistics

Statistical analyses were carried out using appropriate parametric and non-parametric tests according to the distribution of the data. Non-parametric data were expressed as median value and interquartile range, and parametric data as mean and standard deviation. Differences between groups were analyzed by using Student’s *t*-test or Mann–Whitney U, and correlation analyses were performed with Pearson or Spearman tests. Fisher’s exact test was utilized to evaluate categorical data. A *p* value of < 0.05 was considered statistically significant. SPSS 30.0.0 and GraphPad (Version 9.1.2) programs were used as statistical analysis programs.

## 3. Results

### 3.1. Clinical Characteristics and Endovascular Procedures

A total of 56 patients were included in the study and categorized into two groups: those who underwent mechanical thrombectomy alone and those who received bridging therapy consisting of iv tPA and mechanical thrombectomy. In total, 44 (78.6%) patients underwent mechanical thrombectomy, and 12 (21.4%) patients underwent both iv tPA and mechanical thrombectomy, comprising the bridging group. Twenty-one (37.5%) patients were female (Table 1).

In total, 21 (37.5%) patients had occlusion in the ICA, 28 (50%) patients in MCA M1, and 7 (12.5%) patients in the MCA M2 (Table 1). The methods used during mechanical thrombectomy were recorded as stent-retriever in 27 (48.2%) patients, aspiration alone in 16 (28.6%) patients, and the combined method of stent-retriever and aspiration in 13 (23.2%) patients. Successful recanalization was achieved in 48 (85.7%) patients, 37 (84.1%) patients from the MT group, and 11 (91.7%) from the bridging group (Table 2).

According to the TOAST classification, 14 (25%) patients were diagnosed with large artery atherosclerosis, 31 (55.4%) with cardioembolism, 2 (3.6%) with other determined etiologies, and 9 (16.1%) were classified as having cryptogenic stroke (Table 3).

### 3.2. Clinical Outcome

The modified Rankin scale was used to assess clinical outcome. The patients with mRS scores of 0–2 were evaluated as functionally independent. The number of functionally independent patients at 90 days was 24 (42.9%); 20 (45.5%) from the MT group; and 4 (33.3%) from the bridging group. Fifteen (26.8%) patients died at 90 days (Table 2).

In total, 19 (33.9%) patients, 15 (34.1%) in the MT group, and 4 (33.3%) in the bridging group, had intracerebral hemorrhage on follow-up CT imaging.

### 3.3. Thrombus Analysis-H&E Staining

**Figure 1:**
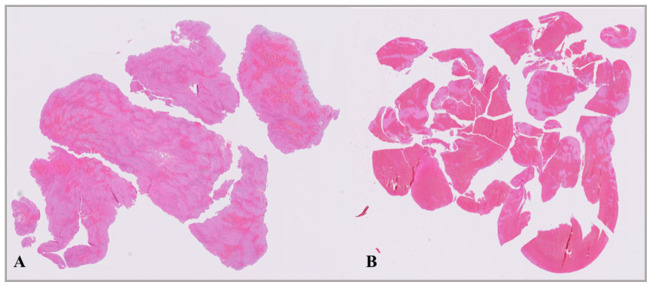
Examples of fibrin-dominant (**A**) and RBC-dominant (**B**) thrombi, H&E staining. (**A**) Representative whole-section image of a fibrin-dominant thrombus retrieved from a patient with M2 occlusion. (**B**) Representative whole-section image of an RBC-dominant thrombus obtained from a patient with M1 occlusion.

All thrombi were stained with hematoxylin–eosin for the identification of fibrin and red blood cells and CD34 for endothelial cells. The thrombi were categorized into two groups: those in which fibrin occupied more than 50% of the area were classified as fibrin-dominant, while those in which red blood cells occupied more than 50% of the area were classified as RBC-dominant (Figure 1). Of all 56 thrombi, 41 (73.2%) were fibrin-dominant, and 15 (26.8%) were RBC-dominant. In the mechanical thrombectomy group, 32 thrombi (72.7%) were fibrin-dominant, 12 (27.3%) were RBC-dominant, whereas 9 thrombi (75%) were fibrin-dominant and 3 (25%) were RBC-dominant in the bridging group. There was no significant difference between treatment groups (*p* = 0.595).

**Figure 2:**
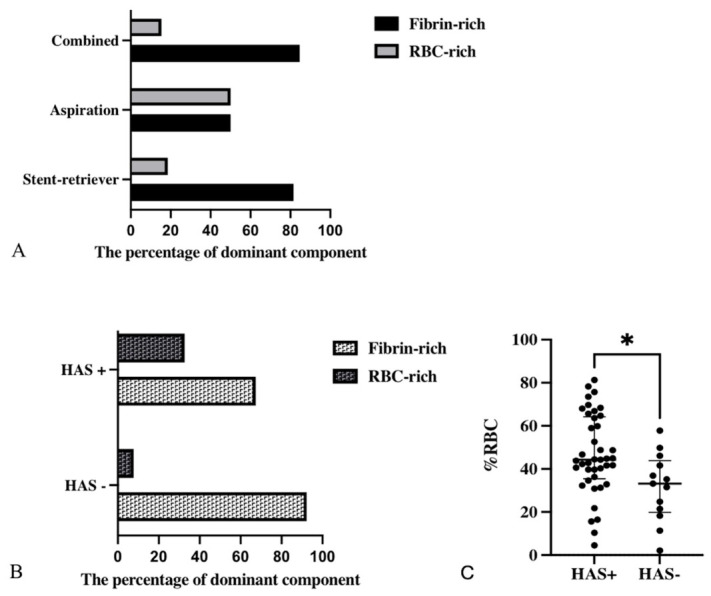
The relationship between thrombus histopathology and the method used during mechanical thrombectomy: fibrin content was higher in thrombi removed with stent-retriever and combined methods (*p* = 0.045) (**A**). The relationship between RBC dominance and hyperdense artery sign: HAS-positive patients had higher RBC dominance than HAS-negative patients (*p* = 0.015, * *p* < 0.05) (**B**,**C**).

The dominant histological component of the thrombus was significantly associated with the location of vessel occlusion (*p* = 0.027). Thrombi retrieved from MCA M2 occlusions demonstrated a higher rate of fibrin dominance compared to those from ICA and M1 occlusions. A significant relationship was also observed between thrombus composition and the mechanical thrombectomy technique used (*p* = 0.045, Figure 2A). Specifically, fibrin content was higher in thrombi removed using stent-retriever or combined methods. However, no significant association was found between thrombus composition and time intervals—symptom-to-door, symptom-to-puncture, and puncture-to-recanalization—number of passes, and recanalization scores (*p* = 0.69, *p* = 0.93, *p* = 0.76, *p* = 0.7, and *p* = 0.24, respectively).

In the fibrin-dominant group, large artery atherosclerosis was identified in 9 (22%) patients, cardioembolism in 23 (56.1%) patients, other determined etiologies in 1 (2.4%) patient, and cryptogenic stroke in 8 (19.5%) patients (Table 3). In the RBC-dominant group, large artery atherosclerosis was observed in 5 (33.3%) patients, cardioembolism in 8 (53.3%) patients, other determined etiologies in 1 (6.7%) patient, and cryptogenic stroke in 1 (6.7%) patient (Table 3). When examining the relationship between stroke etiology and the dominant thrombus component, no statistically significant difference was found (*p* = 0.21, Table 3).

There was a significant association between RBC dominance and the hyperdense artery sign. RBC-dominant thrombi were identified in 14 (32.6%) patients with a hyperdense artery sign, compared to 1 (7.7%) patient without HAS (*p* = 0.015, Figure 2B,C).

### 3.4. Thrombus Analysis—CD34 Staining

**Figure 3:**
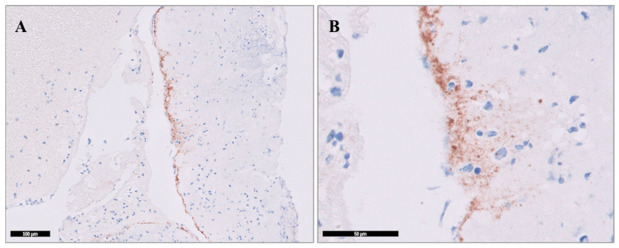
Representative section of a thrombus retrieved from a patient with ICA occlusion, demonstrating CD34 immunohistochemical staining. Endothelial cells are observed on the thrombus surface (**A**) and as endothelial ingrowth extending toward the inner regions of the thrombus (**B**). Images are shown at ×10 and ×40 magnifications.

CD34 staining was performed on 54 thrombi. For analysis, the number of CD34-positive areas within the entire specimen in one section from each thrombus was counted to assess the extent of endothelial cell presence (Figure 3).

The number of areas stained with CD34 was found to be correlated with symptom-to-door and symptom-to-puncture times (r = 0.291, *p* = 0.017, and r = 0.462, *p* < 0.001). There was no such relationship between the duration of the procedure and the number of passes.

No significant association was observed between CD34 positivity and the dominant thrombus component (*p* = 0.12). Similarly, CD34 staining was not significantly associated with stroke etiology (*p* = 0.7).

Upon examination of the thrombi stained with CD34, two distinct staining patterns were observed: surface-only staining in some regions and endothelial cell ingrowth into the thrombus in others (Figure 3). These patterns were assessed separately. A statistically significant relationship was found between the number of areas showing endothelial ingrowth and the site of vessel occlusion (*p* = 0.017). Specifically, thrombi retrieved from more distal occlusions (MCA M2) exhibited fewer CD34-positive areas with endothelial ingrowth compared to those from proximal occlusions (ICA and MCA M1).

## 4. Discussion

This study evaluated histological thrombus composition and endothelial cells within the thrombus in patients with acute ischemic stroke. In our cohort, no significant association was observed between thrombus composition and stroke etiology. Previous studies have reported heterogeneous and sometimes conflicting findings regarding the relationship between thrombus composition and etiological subtypes [9,10,11]. While some reports suggest that cardioembolic thrombi may contain higher fibrin and platelet content and atherosclerotic thrombi may be relatively erythrocyte-rich [4,5], such patterns were not clearly demonstrated in the present data. Similarly, although cryptogenic thrombi have been described as resembling cardioembolic thrombi in certain studies [1,12], our findings did not clearly demonstrate a distinct compositional profile according to etiology.

Another important finding was the association between fibrin dominance in the thrombus and the occlusion site. Thrombi from M2 occlusions were more frequently fibrin-dominant than those from ICA or M1 occlusions. Although not statistically significant, a relatively higher proportion of cryptogenic stroke was observed among patients with M2 occlusions. A similar tendency was noted in the fibrin-dominant group, in which cryptogenic cases were numerically more frequent compared with the RBC-dominant group. This observation would suggest an embolic origin of M2 occlusions, but there was no significant relationship between stroke etiology and fibrin dominance or the location of occlusion. The present data do not permit definitive conclusions regarding the underlying stroke mechanisms, and the observed patterns may, at least in part, reflect sample-related variability. Further studies with larger cohorts are needed to clarify whether these findings represent consistent biological differences.

Fibrin- and RBC-dominant thrombi were comparable between patients treated with mechanical thrombectomy alone and those receiving bridging therapy. These observations do not suggest a measurable difference in thrombus composition at the time of retrieval in relation to prior intravenous tPA administration; however, the sample size and observational design preclude definitive conclusions.

Fibrin-dominant thrombi were retrieved more frequently using stent-retrievers or the combined method rather than aspiration alone, which may result from the greater mechanical resistance of these thrombi. Studies indicate that stent-retrievers are particularly effective at retrieving fibrin-dominant thrombi, which can exhibit higher stiffness and resistance to conventional thrombolytic therapies [13,14,15]. An improved understanding of the dominant thrombus component prior to intervention may potentially inform the selection of thrombectomy techniques.

The hyperdense artery sign may give us an opportunity to visualize a thrombus on a non-contrast CT scan [16,17]. According to our findings, in accordance with the literature, the presence of HAS on baseline CT imaging was significantly associated with RBC-dominant thrombi [1,18,19]. Identifying RBC-dominant thrombi based on HAS carries important clinical implications, particularly for the planning of mechanical thrombectomy. RBC-dominant thrombi exhibit distinct mechanical properties compared with fibrin-dominant thrombi, which may influence their interaction with thrombectomy devices and the success of mechanical thrombectomy [20,21]. As a result, thrombus composition inferred from HAS could help guide the selection of thrombectomy techniques. Given that HAS-negative cases may be more likely to have fibrin-dominant thrombi, the use of a stent-retriever as an initial thrombectomy approach could be considered in these patients, as it may potentially improve procedural success.

This study also evaluated the presence of endothelial cells within thrombi in acute ischemic stroke. An association was observed between the number of CD34-positive areas and symptom-to-door as well as symptom-to-puncture times, which may suggest a potential time-related component in endothelial incorporation. CD34^+^ cells are known to participate in endothelial repair processes following vascular injury and may interact with thrombus surfaces, potentially influencing thrombus organization or remodeling [22,23].

Thrombi retrieved from proximal vessel occlusions exhibited a higher number of CD34-positive areas compared with those from more distal occlusions. This observation may be consistent with greater thrombus–vessel wall interaction in larger proximal arteries such as the ICA and M1 segments. While increased endothelial cell presence could be compatible with differences in thrombus organization, the absence of an association with procedural outcomes suggests that CD34-positive content alone is unlikely to account for mechanical retrievability. Rather, CD34-positive areas may reflect biological variability within thrombi rather than serving as direct indicators of treatment efficacy.

This study has several limitations that should be considered in interpreting the findings. First, the relatively small sample size limits the statistical power and generalizability of the results. Larger cohorts will be necessary to validate these observations. Second, the analysis was restricted to anterior circulation strokes involving ICA, M1, or dominant M2 occlusions. Therefore, the findings may not be applicable to more distal or posterior circulation occlusions, which could demonstrate different histopathological characteristics.

## 5. Conclusions

In this study, associations between thrombus histopathology and selected clinical variables were observed, while no significant relationship was identified with recanalization success or etiology. Although these findings add descriptive data on thrombus composition, their clinical implications remain unclear. The observed association between CD34-positive endothelial cell presence and time intervals may reflect temporal variability in thrombus features. Larger studies are required to further evaluate the potential role of thrombus histopathology in acute ischemic stroke.

## Figures and Tables

**Table 1:** Baseline characteristics of the patients according to treatment strategy.

	MT Group (*n* = 44)	Bridging Group (*n* = 12)	Total (*n* = 56)	*p* Value
**Demographics**				
Age (mean ± SD)	67.8 ± 13.3	60.5 ± 16.6	66.2 ± 14.2	0.17
Female sex (*n*, %)	19 (43.2)	2 (16.7)	21 (37.5)	0.18
**Medical history**				
Diabetes (*n*, %)	15 (34.1)	5 (41.7)	20 (35.7)	0.74
Hypertension (*n*, %)	28 (63.6)	6 (50)	34 (60.7)	0.5
Hyperlipidemia (*n*, %)	23 (52.3)	7 (58.3)	30 (53.6)	0.75
Atrial fibrillation (*n*, %)	22 (50)	3 (25)	25 (44.6)	0.19
Previous stroke (*n*, %)	9 (20.5)	1 (8.3)	10 (17.9)	0.68
Smoking (*n*, %)	6 (13.6)	4 (33.3)	10 (17.9)	0.2
**Clinical features**				
Baseline NIHSS (mean ± SD)	12.9 ± 3.2	10.5 ± 3.1	12.4 ± 3.3	**0.03**
Occlusion				0.3
ICA	17 (38.6)	4 (33.3)	21 (37.5)	
M1	23 (52.3)	5 (41.7)	28 (50)	
M2	4 (9.1)	3 (25)	7 (12.5)	
**Time intervals**				
Symptom-to-door (median, IQR)	108 (50–261)	60 (40–70)	82 (47–212)	**0.039**
Symptom-to-puncture (median, IQR)	312 (195–420)	175 (146–195)	235 (176.5–353)	**<0.001**
Puncture-to-recanalization (median, IQR)	29.5 (20–59)	25 (13.25–50.75)	29.5 (18.5–58)	0.2

**Table 2:** Endovascular procedure metrics and clinical outcomes.

	MT Group (*n* = 44)	Bridging Group (*n* = 12)	Total (*n* = 56)	*p* Value
**Endovascular procedure**				
Technique employed				0.2
Stent-retriever (*n*, %)	20 (45.5)	7 (58.3)	27 (48.2)	
Aspiration (*n*, %)	15 (34.1)	1 (8.3)	16 (28.6)	
Combined (*n*, %)	9 (20.5)	4 (33.3)	13 (23.2)	
Number of passes (median, IQR)	1 (1–3)	1 (1–2)	1 (1–2.75)	0.52
Recanalization score				0.67
TICI 0-2a	7 (15.9)	1 (8.3)	8 (14.3)	
TICI 2b-3	37 (84.1)	11 (91.7)	48 (85.7)	
**Clinical Outcomes**				
NIHSS 24th hour (mean ± SD)	10.4 ± 6	7.5 ± 4.4	9.8 ± 5.8	0.15
NIHSS at discharge (mean ± SD)	8.1 ± 5.5	5.5 ± 4	7.5 ± 5.3	0.19
mRS at 90 days				0.24
mRS 0–2	20 (45.5)	4 (33.3)	24 (42.9)	
mRS 3–5	11 (25)	6 (50)	17 (30.4)	
mRS 6	13 (29.5)	2 (16.7)	15 (26.8)	

**Table 3:** Stroke etiology according to thrombus type, vessel occlusion site, and thrombectomy technique.

**Stroke Etiology and Fibrin/RBC Dominance**				
	**Fibrin-Dominant** **(*n* = 41)**	**RBC-Dominant** **(*n* = 15)**	**Total** **(*n* = 56)**	***p*** **Value**
Large artery atherosclerosis (*n*, %) Cardioembolism (*n*, %) Other etiologies (*n*, %) Cryptogenic stroke (*n*, %)	9 (22) 23 (56.1) 1 (2.4) 8 (19.5)	5 (33.3) 8 (53.3) 1 (6.7) 1 (6.7)	14 (25) 31 (55.4) 2 (3.6) 9 (16.1)	0.29 0.6 0.47 0.24
**Stroke Etiology and Vessel Occlusion Site**				
	**ICA** **(*n* = 21)**	**M1** **(*n* = 28)**	**M2** **(*n* = 7)**	
Large artery atherosclerosis (*n*, %) Cardioembolism (*n*, %) Other etiologies (*n*, %) Cryptogenic stroke (*n*, %)	7 (33.3) 11 (52.4) - 3 (14.3)	6 (21.5) 17 (60.7) 2 (7.1) 3 (10.7)	1 (14.3) 3 (42.9) - 3 (42.9)	0.5 0.55 0.11
**Stroke Etiology and Thrombectomy Technique**				
	**Stent-Retriever** **(*n* = 27)**	**Aspiration** **(*n* = 16)**	**Combined** **(*n* = 13)**	
Large artery atherosclerosis (*n*, %) Cardioembolism (*n*, %) Other etiologies (*n*, %) Cryptogenic stroke (*n*, %)	6 (22) 15 (55.6) 1 (3.7) 5 (18.5)	6 (37.5) 8 (50) 1 (6.3) 1 (6.3)	2 (15.4) 8 (61.5) - 3 (23.1)	0.35 0.94 0.67 0.42

## Data Availability

The original contributions presented in this study are included in the article. Further inquiries can be directed to the corresponding author.

## Abbreviations

The following abbreviations were used in this manuscript.

MT	Mechanical thrombectomy
RBC	Red blood cell
H&E	Hematoxylin–eosin staining
HAS	Hyperdense artery sign
MCA	Middle cerebral artery
NIHSS	National Institutes of Health Stroke Scale
mTICI	Modified thrombolysis in cerebral infarction
mRS	Modified Rankin Scale
TOAST	Trial of ORG 10172 in acute stroke treatment
CT	Computed tomography
ECASS	European Cooperative Acute Stroke Study
ICA	Internal carotid artery
